# Changes in the Organics Metabolism in the Hepatopancreas Induced by Eyestalk Ablation of the Chinese Mitten Crab *Eriocheir sinensis* Determined via Transcriptome and DGE Analysis

**DOI:** 10.1371/journal.pone.0095827

**Published:** 2014-04-22

**Authors:** Yan Sun, Yichen Zhang, Yichen Liu, Shuxia Xue, Xuyun Geng, Tong Hao, Jinsheng Sun

**Affiliations:** 1 Tianjin Key Laboratory of Animal and Plant Resistance, College of Life Sciences, Tianjin Normal University, Tianjin, People's Republic of China; 2 Tianjin Center for Control and Prevention of Aquatic Animal Infectious Disease, Tianjin, People's Republic of China; University of Connecticut, United States of America

## Abstract

**Background:**

To understand the regulation mechanism of eyestalk ablation on the activities of hepatopancreas, Illumina RNA-Seq and digital gene expression (DGE) analyses were performed to investigate the transcriptome of the eyestalk, Y-organ, and hepatopancreas of *E. sinensis* and to identify the genes associated with the hepatopancreas metabolism that are differentially expressed under eyestalk ablation conditions.

**Results:**

A total of 58,582 unigenes were constructed from 157,168 contigs with SOAPdenovo. A BlastX search against the NCBI Nr database identified 21,678 unigenes with an E-value higher than 10^−5^. Using the BLAST2Go and BlastAll software programs, 6,883 unigenes (11.75% of the total) were annotated to the Gene Ontology (GO) database, 7,386 (12.6%) unigenes were classified into 25 Clusters of Orthologous Groups of Proteins (COGs), 16,200 (27.7%) unigenes were assigned to 242 Kyoto Encyclopedia of Genes and Genomes (KEGG) pathways, and1,846 unigenes were matched to “metabolism pathways”. The DGE analysis revealed that 1,416 unigenes were significantly differentially expressed in the hepatopancreas, of which 890 unigenes were up-regulated and 526 unigenes were down-regulated. Of the differentially expressed genes, 382 unigenes were annotated and 63 were classified into metabolism pathways. The results of the real-time polymerase chain reaction (PCR) analysis of four unigenes related to carbohydrate metabolism were consistent with those obtained from the DGE analysis, which demonstrates that the sequencing data were satisfactory for further gene expression analyses.

**Conclusion:**

This paper reported the transcriptom of the eyestalk, Y-organ, and hepatopancreas from *E. sinensis*. DGE analysis provided the different expressed genes of the metabolism processes in hepatopancreas that are affected by eyestalk ablation. These findings will facilitate further investigations on the mechanisms of the metabolism of organic substances during development and reproduction in crustaceans.

## Introduction

The hepatopancreas of crustaceans was described as a metabolic factory, which was the center of lipid and carbohydrate metabolism [1]. The functions of the hepatopancreas are considered to include the storage and depletion of organic substances to support various significant life activities, such as ovarian maturation, vitellogenin synthesis and molting [2]–[4].

Murugesan [5] reported the effect of eyestalk ablation on the biochemical composition of the crab, *Charybdis lucifera*, the result showed that the content of carbohydrate, lipid, fatty acids and protein in crabs of eyestalk ablation were higher than intact crabs, eyestalk ablation can cause the glycogen synthesis [6] in hepatopancreas. However, the exact regulation mechanism of eyestalk ablation on the activities of hepatopancreas is not clear. Some studies revealed that when the hepatopancreas was incubated with CHH; the rate of glucose into glycogen was decreased [7]. These results suggest a physiological role for CHH in the control of carbohydrates and lipid metabolism in crustacean. Crustacean hyperglycemic hormone (CHH), a neuropeptide synthesized and secreted from the X-organ/sinus gland (XO/SG) complex in the eyestalk, plays a central role in the regulation of energy metabolism [8]. The hepatopancreas is the primary tissue on which CHH act [9]. A number of studies have reported that CHH has the function to elevate the glucose level in the hemolymph of many decapods [10]–[12]. Besides the researches on the macro level, some genes related to glycogen metabolism, such as glycogen phosphorylase, glycogensynthase, fructose1, 6-bisphosphatase and phosphoenolpyruvate carboxykinase were also studied on mRNA level [13]. And the results confirmed that these genes were involved in glycogen metabolism in hepatopancreas, and were regulated by CHH. The release of free fatty acids and phospholipids from *Orconectes limosus* hepatopancreas in vitro was significantly increased in the presence of CHH [14]. Above results indicated that the absence of CHH may be the key reason why organics metabolism changed after eyestalk ablation.

In this paper, we analyzed the transcriptome of the eyestalk, Y-organ, and hepatopancreas from *Eriocheir sinensis*, one of the most economically aquaculture species in China, using the Illumina RNA-Seq method [15]. This method has been used in transcriptome profiling studies in many species [16]–[20]. Based on the transcriptome results obtained, a digital gene expression (DGE) analysis, which sequences the short tags generated by end nucleases from the 3′ ends of genes (the copy number of each tag indicates the expression level of the corresponding gene) [21], was performed to investigate the metabolism-related genes that were differentially expressed under the eyestalk ablation challenge in the hepatopancreas of *E. sinensis*. The sequencing results can provide new insights that will help reveal the molecular mechanism of the metabolism of organic substances regulated by eyestalk ablation.

## Materials and Methods

### Preparation of tissue samples

The healthy juvenile Chinese mitten crabs (body weight 5–6 g), with good vitality were purchased from the Tianjin Fisheries Institute, and then acclimatized in freshwater at 18–20°C (photoperiod L12:D12) for seven days. Intermolt crabs were used for the experiments. Three types of tissues, including the eyestalk, Y-organ, and hepatopancreas, were separated and collected. All of the samples were immediately frozen in liquid nitrogen and stored at −80°C until use.

### RNA isolation and transcriptome sequencing

The total RNA from the three tissues (eyestalk, Y-organ, and hepatopancreas) was extracted using the TRIzol method (Invitrogen, USA) according to the manufacturer's protocol. Equal quantities of RNA from each tissue were then pooled for transcriptome analysis. The samples for transcriptome analysis were prepared using an Illumina kit following the manufacturer's instructions. Briefly, beads with Oligo (dT) were used to isolate the poly (A) mRNA. Fragmentation buffer was added to interrupt the mRNA strands into short fragments. Using these short fragments as templates, a random hexamerprimer was used to synthesize the first-strand cDNA. The second-strand cDNA was synthesized using buffer, dNTPs, RNaseH, and DNA polymerase I (Invitrogen, USA). The short fragments were purified with a QiaQuick polymerase chain reaction (PCR) extraction kit and resolved with EB buffer for end repair. The short fragments were then connected with sequencing adapters. After agarose gel electrophoresis, the suitable fragments were selected for use as templates for PCR amplification. Finally, the library was sequenced using Illumina HiSeq™ 2000.

### Analysis of transcriptome assembly results

The cDNA library was sequenced on Illumina HiSeq™ 2000 sequencing platform and the 90 bp raw reads were cleaned by removing adaptor sequences, empty reads and low quality sequences. The clean reads were submitted to EBI European Nucleotide Archive (ENA) database (http://www.ebi.ac.uk/ena/data/view/PRJEB4541, accession number: PRJEB4541). The transcriptome *de novo* assembly was performed using the short reads assembling program Trinity [22]. Trinity first combines those reads with a certain length of overlap to form longer fragments, which are called contigs. The reads are then mapped back to the contigs. This process is able to detect contigs from the same transcript as well as the distances between these contigs and paired-end reads. Finally, Trinity connects the contigs and obtains sequences that cannot be extended on either end. Such sequences are defined as unigenes. When multiple samples from the same species are sequenced, the unigenes from each sample's assembly can be further analyzed through sequence splicing and the removal of redundancy using sequence clustering software to acquire non-redundant unigenes with the longest length possible. Unigenes with a minimum length of 200 bp were selected and submitted to EBI European Nucleotide Archive (ENA) database (http://www.ebi.ac.uk/ena/, accession number: HAAX01000001-HAAX01058228). In the final step, a BlastX alignment (E-value<0.00001) between the unigenes and protein databases, such as Nr, Swiss-Prot, Kyoto Encyclopedia of Genes and Genomes (KEGG), and Clusters of Orthologous Groups of Proteins (COG),is performed, and the best-aligned results are used to decide the sequence direction of the unigenes. If the results of different databases conflict, a priority order of Nr, Swiss-Prot, KEGG, and COG should be followed in the decision of the sequence direction of the unigenes. If a unigene is not aligned to any of the above mentioned databases, its sequence direction will be decided using a software program named ESTScan [23].

The unigenes were first aligned by BlastX to the Nr protein database with a significant threshold E-value of 10^−5^. The Gene Ontology (GO) annotation of the unigenes was obtained through the Blast2GO program [24] with an E-value cut-off at 10^−5^. After obtaining the GO annotation for all of the unigenes, the WEGO software [25] was used to perform a GO functional classification for all of the unigenes and to understand the distribution of the functions of all of the genes in the species at the macro level. COG and KEGG pathway annotation was achieved using the BlastAll software (E-value<10^−5^) against the COG database [26] and KEGG database [27], respectively.

### Digital gene expression library preparation and sequencing

A total of 10 crabs that were selected randomly from the rearing animals in the laboratory were equally divided into two groups. The group that had its eyestalks removed was the experimental group (A1), and the other group, which did not receive any treatment, was the control group (A0). The hepatopancreas from the crabs of these two groups were separated after 48 h, and the dissected tissues were immediately preserved in liquid nitrogen for RNA extraction. After extracting the total RNA from the two samples, the generated cDNA library was sequenced using Illumina HiSeq^TM^2000. The original image data were transferred into sequence data through base calling.

### Analysis of digital gene expression tags

To obtain the clean reads, all raw reads were filtered to remove low-quality reads (the percentage of the low-quality bases of quality value ≤5 is more than 50% in a read) as well as reads with adaptors or reads in which unknown bases are more than 10%. Then, clean reads were mapped to reference sequences using SOAPaligner/soap2 [28]. Only mismatches with one or two bases were allowed in the alignment. The gene expression level is calculated by using RPKM (reads per kb per million reads) [29]. A statistical analysis of the frequency of each read in two different cDNA libraries was further performed to screen the differentially expressed genes in hepatopancreas of *E. sinensis* depending on whether the eyestalks ablated or not. We used FDR≤0.001 and an absolute value of log_2_ratio ≥1 as the threshold to judge the significance of the gene expression difference. Finally, the identified genes of different expression levels were mapped to terms in the GO and KEGG databases.

### Quantitative real-time PCR (qRT-PCR) validation

To validate the DGE results, four annotated unigenes that were found to be up-regulated in the DGE analysis compared with the control and found to have a relationship with carbohydrate metabolism were selected to be analyzed using real-time PCR; the unigene number, predicted gene name, and specific primers used are listed in Table 1. The eyestalks of 6 crabs (carapace width: 4–5 cm) were ablated as described in the DGE library preparation section. The hemolymph of the crabs was collected at 0 and 48 h and centrifuged at 800×g for 10 min at 4°Cin a refrigerated centrifuge. A glucose measurement kit (Nanjingjiancheng, China) was then used to evaluate the concentration of glucose in the serum. The hepatopancreas of these crabs was also collected at the same time points immediately after collection of hemolymph. The total RNA of each hepatopancreas was extracted using the TRIzol method (Invitrogen, USA) as described above. The cDNA was synthesized using the Superscript III first-strand synthesis system (Invitrogen, USA) according to the manufacturer's protocol. The real-time PCR analysis was performed using an IQ5 system (Bio-Rad, USA) with a SYBR PrimeScript RT-PCR kit (TaKaRa, Japan) according to the manufacturer's instruction. The expression levels of each gene were normalized to β-actin (β-actin-f: 5′-ATCGTGCGAGACATCAAGGA-3′; β-actin-r: 5′-AGGAAGGAAGGCTGGAAGAGT-3′). All of the real-time PCR experiments were performed in three biological replicates and the average threshold cycle (Ct) was calculated with the 2^−ΔΔCt^ method [30]. The relative expression quantification of the target genes in A0 and A1 groups were statistically tested by ANOVA followed by T-test (P<0.05).

**Table 1:** The unigenes for validation of DGE analysis and real-time PCR primers.

Unigene Number	similarity	Real-time PCR Primer sequence (5′-3′)
**33700**	**Kexokinase**	**F: GCGGAACTTGAAACTGGG**
		**R: ATCGCCGACACCGTAATG**
**19969**	**Triosephosphate isomerase**	**F: CTCAGATGAAGGCTTTGGTCC**
		**R: TGACATTATCTCGCAGCCACT**
**19080**	**Pyruvate kinase3**	**F:CACGGACAAGGAGTTCTACGA**
		**R:GCGATGAGCGAGATGAGCC**
**46238**	**UDP-glucose4-epimerase**	**F: TACAGGGCAGGGAGTTTCAGT**
		**R: CCTTCACGACGAGGTTTCAGC**

## Results

### Transcriptome data and functional distribution

**Figure 1:**
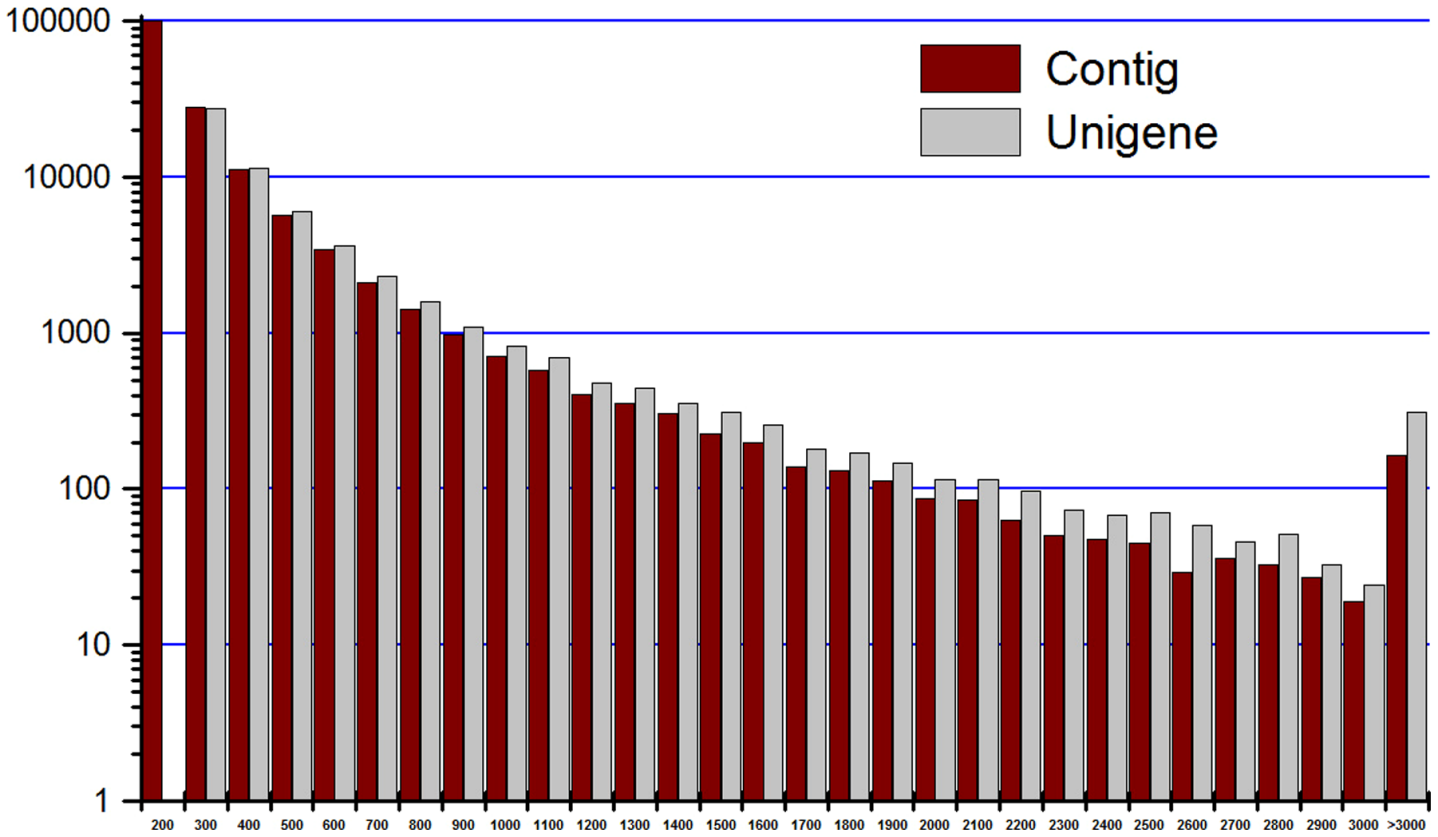
Statistics of the size distributions of contigs and unigenes. (X-axes indicates the sequence size (nt), Y-axes indicates the number of assembled contigs and unigenes.).

To construct the *E. sinensis* transcriptome data, sequences of mRNA pooled from the eyestalks, Y-organs, and hepatopancreas of *E. sinensis* were analyzed using the Illumina HiSeq^TM^2000 method. A total of 26,208,092 clean reads (the number of total clean nucleotides was 2,358,728,280 nt; the Q20 and GC percentages were 96.68% and 45.08%, respectively) were obtained. From these clean reads, 157,168 contigs were assembled, and 58,582 unigenes (http://59.67.75.245/college/skxy/skin/one/show1.asp?id=2066), including 1,522 distinct clusters and 57,060 distinct singletons, were constructed from these contigs with SOAPdenovo. The statistical results obtained from the *E. sinensis* transcriptome sequencing and assemblies are listed in Table 2, and the size distributions of the contigs and unigenes are shown in Fig. 1.

**Table 2:** Statistics of *E.sinensis* transcriptome sequencing and assembly.

Name	Number of sequences	Mean length (nt)
**Total raw reads**	**30,116,012**	**-**
**Total clean reads**	**26,208,092**	**-**
**Total clean nucleotides (nt)**	**2,358,728,280**	**-**
**Contigs**	**157,168**	**236**
**Unigenes**	**58,582**	**459**

The unigene sequences were first aligned against the Nt, Nr, and Swiss-Prot databases using BlastN and BlastX searches with an E-value less than10^−5^. This analysis indicated that 21,678 unigenes (37.00% of the total unigenes) were matched to the Nr database (Table S1), 11,823 unigenes (20.18% of the total unigenes) were matched to the Nt database (Table S2), and 18,446 unigenes (31.49% of the total unigenes) were matched to the Swiss-Prot database (Table S3). However, 33,374 unigenes (56.97% of the total unigenes) could not be matched to any genes included in these three databases. The unigenes that could not be aligned to any database were scanned by ESTScan to determine the nucleotide sequence direction and amino sequence of the predicted coding region. The Protein Coding Region Prediction (CDS) of 5,372 unigenes (0.092% of the total unigenes) were successfully predicted by ESTscan.

**Figure 2:**
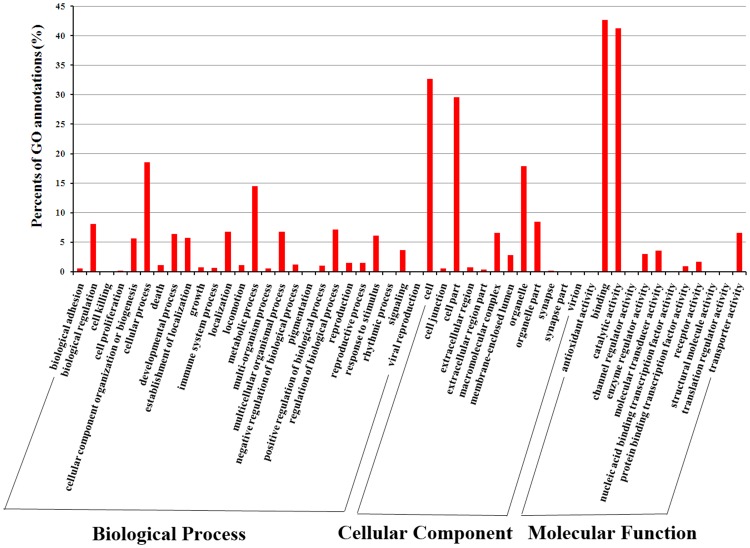
GO categories of the unigenes. 6883 unigenes were assigned 50 GO annotations, which were divided into three categories: biological processes, cellular components, and molecular functions.

With the Nr annotation, the Blast2GO program was used to obtain the GO annotation of the unigenes. After obtaining the GO annotation for all of the unigenes, we used the WEGO software to perform a GO functional classification for all of the unigenes and to understand the functional distribution of the species at the macro level. Of the 58,582 unigenes in *E. sinensis*, 6,883 unigenes (11.75% of the total) were annotated to the GO database with confident matches. Of these, 17,184 unigenes were assigned to the biological process category, 12,502 were assigned to the cellular component category, and 6,397 were assigned to the molecular function category. The unigenes in the biological process category were divided into 26 different biological processes, of which the cellular process (3,191 unigenes; 18.5% of the total unigenes) and the metabolic process (2,492; 14.5%) were the most highly represented. Other processes, such as biological regulation (1,392; 8.1%), the developmental process (1,094; 6.37%), localization (1,166; 6.79%), the multicellular organismal process (1,170; 6.81%), the regulation of the biological process (1,228; 7.15%), and the response to stimulus (1,057; 6.15%), were also represented. A total of 12 GO functional groups were assigned to the cellular component category, of which the cell (4,086; 32.69%) and the cell part (3,691; 29.52%) were the most highly represented, and 12 GO functional groups were assigned to the molecular function category, of which binding (2,727; 42.63%) and catalytic activity (2,638; 42.24%) were the most highly represented (Fig. 2).

**Figure 3:**
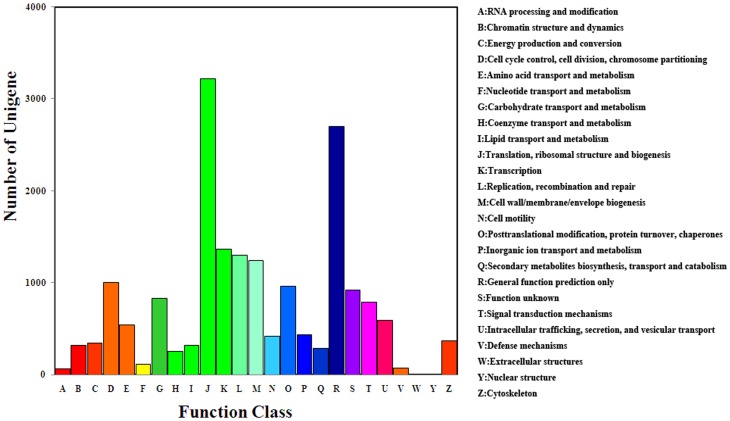
COG Classification of the unigenes. 7386 unigenes were classified into 25 COG categories.

The unigenes obtained were aligned to the COG database to predict and classify their possible functions. It was found that 7,386 of the total unigenes have a COG classification, and these unigenes were classified into 25 categories. Of these categories, the cluster for “translation, ribosomal structure, and biogenesis” represents the largest group (3,215 unigenes), followed by “cell cycle control, cell division, and chromosome partitioning” (1,004 unigenes), “transcription” (1,366 unigenes), “replication, recombination, and repair” (1,298 unigenes), and “cell wall, membrane, and envelope biogenesis” (1,241 unigenes), whereas “nuclear structure” (six unigenes) and “extracellular structures” (10 unigenes) represent the smallest groups. In addition, 2,698 and 919 unigenes were assigned to the “general function prediction only” and “function unknown” categories, respectively. Thus, it was not possible to predict the functions of most of the unigenes assigned through the COG database (Fig. 3).

To identify the biological pathways that are active in *E. sinensis*, 58,582 unigenes were matched to the reference canonical pathways in KEGG, which is a database that is able to analyze the gene product during a metabolism process and the related gene function in the cellular processes. A total of 16,200 unigenes were assigned to 242 pathways. The pathways with the most representation were “metabolic pathways” (1,846 unigenes; 11.4%), “regulation of the actin cytoskeleton” (770 unigenes; 4.75%), “RNA transport” (565 unigenes; 3.49%), “spliceosome” (550 unigenes; 3.4%), and “pathways in cancer” (500 unigenes; 3.09%). Other important pathways that are related to the growth and energy metabolism include the “MAPK signaling pathway” (373 unigenes; 2.3%), “glycolysis/gluconeogenesis” (110 unigenes; 0.68%), “fatty acid metabolism” (101 unigenes; 0.62%), the “PPAR signaling pathway” (91 unigenes; 0.56%), and “steroid biosynthesis” (16 unigenes; 0.1%).

The results obtained from thesubjection of the assembled unigenes to BLAST searchesagainst the GO, COG, and KEGG databases are presented in Table 3.

**Table 3:** Summary of annotation results in *E.sinensis*.

Name of database	NR	NT	SwissPort	KEGG	COG	GO	ALL
**Number of Unigene**	**21,678**	**11,823**	**18,446**	**16,200**	**7,386**	**6,883**	**25,208**

### DGE profiling of differential gene expression with and without eyestalk ablation

Two cDNA libraries derived from two hepatopancreas samples that were separated from the normal crabs (A0) and the crabs without eyestalks (A1) were constructed and sequenced using Illumina HiSeq^TM^2000. A total of 6,026,027 and 5,849,598 clean reads were generated from the A0 and A1 samples, respectively, after the dirty raw reads were filtered out. The clean reads were mapped to the *E. sinensis* transcriptome (58,582 unigenes) using SOAPaligner/soap2, and only mismatches of one or two bases were allowed in the alignment. A total of 4,352,609 and 4,149,746 reads derived from the A0 and A1 samples, respectively, matched unigenes in the reference database, of which 3,252,460 and 3,105,718 reads in the A0 and A1 samples, respectively, were perfect matches. In addition, 3,735,346, 617,263, and 1,673,418 of the total reads derived from the A0 samples were unique matches, multi-position matches, and total unmapped reads, respectively, and 3,465,765, 683,981, and 1,699,852 of the total reads derived from the A1 samples were unique matches, multi-position matches, and total unmapped reads, respectively. The data are presented in Table 4.

**Table 4:** Statistics of DGE sequencing data from *E. sinensis* (A0 and A1).

	Total Reads	Total BasePairs	Total Mapped Reads	Perfect Match	Unique Match	Multi-position Match	Total Unmapped Reads
**A0**	**6,026,027**	**2.95E+08**	**4,352,609**	**3,252,460**	**3,735,346**	**617,263**	**1,673,418**
**A1**	**5,849,598**	**2.87E+08**	**4,149,746**	**3,105,718**	**3,465,765**	**683,981**	**1,699,852**

To identify those genes that exhibit a significant change in expression in the hepatopancreas of *E. sinensis* upon eyestalk ablation, the differentially expressed tags between the A0 and A1 groups were detected based on the criteria of significance (FDR≤0.001 and log_2_ratio≥1). As a result, there were 1,416 unigenes that were significantly differentially expressed in the hepatopancreas, of which 382 are annotated and the remainders are not characterized. After the statistical analysis, 890 and 526 unigenes were found to be up-regulated and down-regulated, respectively (Table S4).

The 1,416 differentially expressed unigenes were compared to the GO database, and the result revealed that 136 unigenes are involved in the cellular component, 185 are involved in molecular function, and 164are involved in the biological process. In addition, 88 unigenes were classified in all three categories. In the cellular component category, a large number of unigenes were localized in the “intracellular” (106; 77.9%), “intracellular part” (103; 75.7%), “cell” (131; 96.3%), and “cell part” (131; 96.3%) components, whereas most unigenes were assigned to “catalytic activity” (128; 69.2%) and “binding” (122; 65.9%) in the molecular function category. A great number of the unigenes included in the biological process category were matched to the “metabolic process” (113; 68.9%) and other biological process related to metabolism, such as “cellular amino acid metabolic process”, “protein metabolic process”, and “cellular carbohydrate metabolic process”.

To assess the functions of the genes that are differentially expressed between the A0 and A1 groups, all of the genes were mapped to the KEGG database and compared with the entire transcriptome dataset. The comparison results indicated that 291 unigenes were assigned to 163 KEGG pathways. Of these, 50 pathways were related to the metabolic process (approximately 63 unigenes were classified in metabolism pathways), and the metabolism pathways were mainly related to carbohydrates, lipids, and amino acids, e.g., “glycolysis/gluconeogenesis”, “fructose and mannose metabolism”, “galactose metabolism”, “fatty acid biosynthesis”, “fatty acid metabolism”, and various types of amino acid metabolism pathways. This result is similar to that obtained in the biological process category through the GO annotation. A large number of differently expressed genes, such as kexokinase, triosephosphateisomerase, pyruvate kinase, glycogen phosphorylase, α-glucosidase, and α-amylase, were included in the carbohydrate metabolism pathways, whereas acetyl-CoA carboxylase, fatty acid synthase, long-chain fatty acid CoA ligase, and pancreatic triacylglycerol lipase were included in the lipid metabolism pathway, and glutamine synthetase, D-3-phosphoglycerate dehydrogenase, and glycine N-methyltransferase were included in the amino acid metabolism pathway (Table 5).

**Table 5:** Statistics of mainly different expression genes from *E. sinensis* (A0 and A1) mapped to metabolism pathways.

	Unigene Number	Predict name	Fold change	Pathway ID
**Carbohydratesmetabolism**	**33700**	**Kexokinase**	**1.5**	**ko00500;ko00052;ko00520;ko00010;ko00051**
	**19969**	**Triosephosphate isomerase**	**1.2**	**ko00010;ko00051**
	**19080**	**Pyruvate kinase3**	**1.2**	**ko00010**
	**46238**	**UDP-glucose4-epimerase**	**2.0**	**ko00052;ko00520**
	**20596**	**Membrane-bound trehalase**	**1.1**	**ko00500**
	**21089**	**Trehalose-6-phosphate synthase**	**1.4**	**ko00500**
	**19292**	**Glycogen phosphorylase**	**1.1**	**ko00500**
	**20718**	**Zinc-dependent alcohol dehydrogenase**	**−1.5**	**ko00010**
	**42872**	**α-glucosidase**	**−1.0**	**ko00500;ko00052**
	**43023**	**Glycosyltransferase**	**−1.4**	**ko00500**
	**48626**	**α-amylase**	**−1.1**	**ko00500**
**Lipids metabolism**	**16163**	**Acetyl-CoA carboxylase-like**	**1.2**	**ko00061**
	**19548**	**Fatty acid synthase**	**1.2**	**ko00061**
	**13687**	**Long-chain fatty acid CoA ligase**	**−1.9**	**ko00071**
	**20446**	**Pancreatic triacylglycerol lipase-like**	**−1.7**	**ko00561**
**Amino acids metabolism**	**20688**	**Glutamine synthetase**	**−1.8**	**ko00250**
	**19318**	**D-3-phosphoglycerate dehydrogenase**	**−4.1**	**ko00260**
	**6453**	**Cystathionine γ-lyase**	**−1.3**	**ko00260;ko00270**
	**1598**	**Glycine N-methyltransferase-like**	**−2.0**	**ko00260**
	**18852**	**Hemocyanin subunit**	**−1.2**	**ko00350**
	**49719**	**Glutathione S-transferase**	**−1.2**	**ko00350;ko00480**
	**28622**	**3-hydroxyanthranilate 3,4-dioxygenase**	**−1.4**	**ko00380**

*Ko00010:Glycolysis/Gluconeogenesis; Ko00051:Fructose and mannose metabolism; Ko00052:Galactose metabolism; Ko00500:Starch and sucrose metabolism; Ko00520:Amino sugar and nucleotide sugar metabolism; Ko00061:Fatty acid biosynthese; Ko00071:Fatty acid metabolism; Ko00561:Glycerolipid metabolism; Ko00250:Alanine, aspartate and glutamate metabolism; Ko00260:Glycine, serine and threonine metabolism; Ko00270:Cysteine and methionine metabolism; Ko00350:Tyrosine metabolism; Ko00380:Tryptophan metabolism; Ko00480:Glutathione metabolism.

### Validation of gene expression by real-time PCR

**Figure 4:**
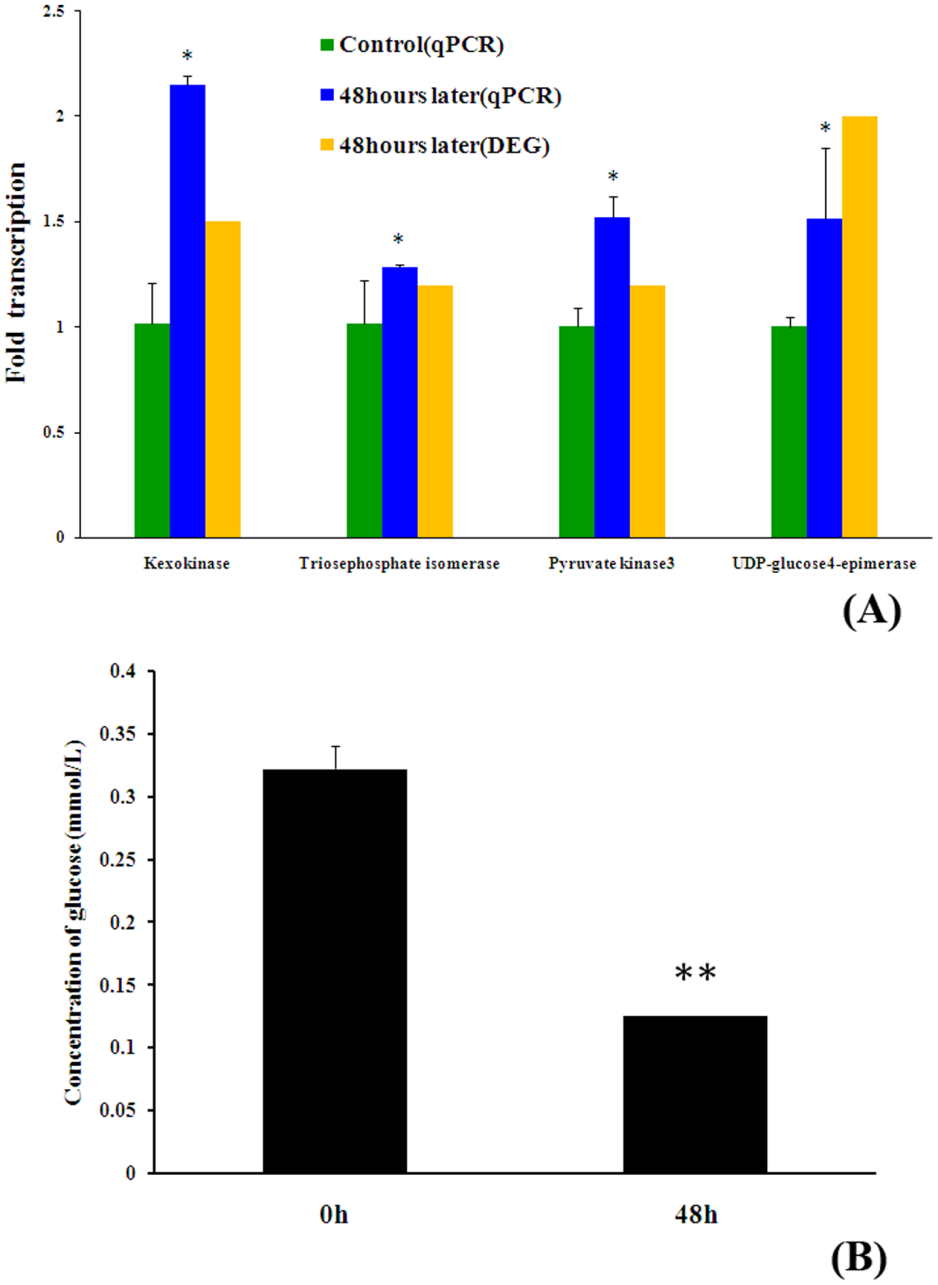
Validation of the DGE analysis. (A): 4 assembled unigenes related to carbohydrate metabolism were selected for Real-time PCR validation of the relative expression levels of transcripts selected from the DGE analysis. (B): Glucose concentration in hemolymph after eyestalk ablation at 0 and 48 hours. Experiments were performed three times with similar results. Bars display mean+SEM, and statistical analysis was performed using Student's T test. **, P<0.01; *, P<0.05.

The results of the real-time PCR analysis indicated that the four genes encoding kexokinase, triosephosphate isomerase, pyruvate kinase, and UDP-glucose4-epimerase, which were annotated as significant enzymes with a biological function associated with the carbohydrate metabolism, were highly expressed in the hepatopancreas of crabs two days after the induction of eyestalk ablation (48 h) compared to the control crabs. The results exhibit trends that are similar to those obtained with the DGE analysis (Fig. 4A). The concentration of glucose in the serum was lower in the experimental group compared with the control group (0 h) 48 h after the induction of eyestalk ablation, as shown in Fig. 4B. As expected, the trends obtained for the four unigenes contrasted the results of the glucose concentration, which indicates that the data derived from the DGE analysis was satisfactory for further gene expression analysis.

## Discussion

In this study, we used next-generation sequencing approach to investigate the transcriptome of the eyestalk, Y-organ, and hepatopancreas of *E. sinensis*. A total of 58,582 unigenes were assembled from 157,168 contigs. Of these unigenes, 25,208 were new transcripts that were not matched to any database (Nt, Nr, and Swiss-Prot). This finding provides a good foundation for further research on the discovery of new genes and facilitates the understanding of the genome background of crustaceans. The possible functions of the assembled unigenes were analyzed through their matching to the KEGG database, and the results indicated that the largest number of unigenes were included in metabolism processes. This finding indicates that the eyestalk, Y-organ, and hepatopancreas of this crab participate in the synthesis and decomposition of substances that are needed for the growth and development of the crab. A large number of unigenes were not matched to any pathways, possibly because the KEGG database mainly record the signaling pathways of model organisms, the genomic background of crustaceans are not clearly understood, and some of the unigenes were found to be unique to crab. However, we obtained some useful pathways from the KEGG analysis that have not been previously reported in crustaceans, such as the “GnRH signaling pathway”, “progesterone-mediated oocyte maturation”, “steroid hormone biosynthesis”, and “insect hormone biosynthesis”. Thus, these pathways might provide a reference for the future construction of the signaling pathways related to hormone synthesis and reproduction that belong uniquely to crustaceans.

The function of CHH secreted from the eyestalk of crustaceans is hypothesized to be associated with the regulation of the glucose levels in the hemolymph [31]. In addition, large amounts of organic resources are accumulated in the hepatopancreas of crustaceans; such stores are mobilized to meet the demands of events, such as molting, growth, and reproduction [32]. Thus, we analyzed the genes that are differentially expressed in the hepatopancreas in response to the challenge of eyestalk ablation using the DGE method based on the results from the transcriptome analysis. The results indicated that a total of 1,416 unigenes are significantly differentially expressed in the hepatopancreas, of which 890 are up-regulated and 526 are down-regulated. Of these 1,416 unigenes, 382 were identified, including 119 that were up-regulated and 263 that were down-regulated. The KEGG analysis indicated that most of the annotated DEGs were enriched in carbohydrate metabolism processes, such as “glycolysis/gluconeogenesis”, “fructose and mannose metabolism”, “galactose metabolism”, and “starch and sucrose metabolism”. This finding indicated that CHH may have a function associated with the regulation of carbohydrate metabolism. Many of the differentially expressed genes that were enriched in the pathways of multiple carbohydrate metabolisms were key enzymes that regulated the interconversion of carbohydrates in response to changes in the external environment and physiology. For example, kexokinase, pyruvate kinase, triosephosphateisomerase, and UDP-glucose4-epimerase, which were enriched in the glycolysis/gluconeogenesis and multiple carbohydrate metabolism pathways, were all up-regulated, whereas α-glucosidase and α-amylase were down-regulated. It suggested that eyestalk ablation inhibit the processes of starch and glycogen translated into glucose and cause the accumulation of starch and glycogen in hepatopancreas. Thus, we summarized that the genes related to the accumulation of carbohydrate process were up-regulated; and the genes related to the consumption of carbohydrate process were down-regulated. The changes in the expression of these genes indicate that eyestalk ablation causes the accumulation of carbohydrate in hepatopancreas [33], [34] and that the expression of these genes is probably regulated by CHH. In addition to the carbohydrate metabolism pathways, eyestalk ablation also caused changes in the lipid and protein metabolisms. In the lipid metabolism, the unigenes that were matched to the pathway of fatty acid synthesis were all up-regulated, whereas the unigenes annotated to the fatty acid metabolism pathways were all down-regulated. A similar result was obtained with protein metabolism: the unigenes annotated to the amino acid metabolism pathway were all down-regulated. It illustrated that eyestalk ablation can cause the accumulation of carbohydrate; lipid and protein and inhibit the processes of organics consumption in hepatopancreas of *E. sinensis*. Thus, eyestalk ablation inhibited the metabolic rate of lipids and proteins, induced the synthesis of lipids and proteins, decreased the blood glucose level, and ultimately increased the storage of energy. From the DGE analysis results, we filtered the genes associated with metabolism processes that were affected by eyestalk ablation (Table 5) and speculated that the CHH-dependent regulation of the glucose levels may depend on these differentially expressed genes. The expression of genes related to the synthesis of glycogen, fatty acids, and amino acids increased in response to eyestalk ablation, whereas the expression of the genes related to the depletion of organic substances decreased when endogenous CHH was removed, which is why eyestalk ablation results in a decrease in the glucose levels in the hemolymph and energy accumulation in the hepatopancreas.

In conclusion, we have generated a transcriptome profile of *E. sinensis* expressed in eyestalk, Y-organ, and hepatopancreas using mRNA sequencing. These profiles will be useful for understanding the genome background of crustaceans and will promote studies on the discovery of functional genes. The results of the DGE analysis suggest that eyestalk, which likely through the neuropeptide CHH regulates not only the carbohydrate metabolisms but also the lipid and protein metabolisms in the Chinese mitten crab. Thus, the genes related to the metabolism processes that are differentially expressed under the challenge of eyestalk ablation were filtered, and the findings probably provide insights into the molecular mechanisms of the metabolism processes affected by the secretion of CHH from the eyestalk and will facilitate further research on the mechanisms of energy conversion during molting, development, and reproduction in crustaceans.

## Supporting Information

Table S1 BlastX searching of the unigenes against NCBI Nr database.
(XLSX)

Table S2 BlastX searching of the unigenes against NCBI Nt database.
(XLSX)

Table S3 BlastX searching of the unigenes against Swissprot database.
(XLSX)

Table S4 The significant up-regulated and down-regulated unigenes between A0 and A1.
(XLSX)
